# Mr. Howard Marsh on Infective Arthritis

**Published:** 1902-12-20

**Authors:** 


### MR. HOWARD MARSH ON INFECTIVE ARTHRITIS.

In the Bradshaw Lecture, delivered before the Royal College of Surgeons, Mr. Howard Marsh dealt with the subject of infective arthritis, showing that while it has long been known that in such instances as pyæmia and other allied conditions the joints are liable to become infected, recent advances in bacteriology and exact clinical observation have made it clear that joint affections of infective origin are much more frequent than was formerly supposed. It is now well established that inflammation of the peritoneum is in the large proportion of cases infective and due to micro-organisms, and as it is with the peritoneum so it is with the joints. Inflammation in a joint is never idiopathic and is often infective, being produced by agencies which, when they were first discovered, were supposed to be limited in their action to other structures. A notable illustration of this is met with in the case of the pneumococcus, which, besides being the pathogenic agent concerned in the production of lobar pneumonia, has been found to have a far wider range, producing pleurisy, peritonitis, pericarditis, meningitis, and also primary arthritis. Thus, in addition to the common forms of septicæmic and gonococcal infection we have that of the pneumococcus, typhoid fever, influenza, scarlet fever, dysentery, erysipelas, and glanders, and lastly the doubtful case of acute rheumatism. This, however, does not entirely cover the field, for as Mr. Marsh points out, in addition to these instances, in which this condition occurs in connection with various specific diseases, cases occur in which joint affections arise in connection with pyrexia, the nature of which is uncertain ; cases which are traditionally regarded as examples of acute or subacute rheumatism. The term "rheumatism," however, has been made to cover a broad expanse of loose pathology, and to-day, when rheumatism itself has been shown to be probably an infective disease, it is seen that many cases which have been termed rheumatism may also belong to the infective class, such infections being due to various known and probably to many other as yet unknown forms of microbic life. Scattered about in everyday practice are a number of cases which have hitherto been regarded as rheumatic but which are really infective, and although such cases taken singly may present no distinct resemblance to the typical forms of infective arthritis, yet when they are massed together and studied in connection with observations recently made in bacteriology their nature appears to admit of hardly a doubt.

What, then, are the changes produced in the joints by infective arthritis? In some they are slight and transient and result from synovitis with but only a limited amount of serous effusion into the cavity of the joints, as in the arthritis which occurs in the early stage of scarlet fever and typhoid fever. In a second group one or more joints are painful and swollen from effusion of fluid into the synovial membrane ; these cases closely simulate acute rheumatism, but although the effused fluid is at first merely turbid, it in some cases soon becomes purulent. In the third group of cases the inflammatory process chiefly involves the periarticular tissues, leading to brawny or boggy swelling and to reddening of the skin, which becomes so stretched and shiny as to suggest the presence or near approach of suppuration. This is the type with which we have long been familiar in some cases of gonorrhœal infection. In this form, however, there is frequently no effusion in the cavity of the joint, and though it seems imminent, suppuration rarely occurs. But these cases run a tedious course, are often attended with much pain, and show a strong tendency to result in fibrous ankylosis or even complete synostosis. A fourth group includes those in which the arthritis is from the first acute and destructive, suppuration and burrowing of pus taking place early, a condition seen in its most marked form in pneumococcic arthritis.

In the first class of cases the prognosis is favourable, and the only treatment required will be a suitable splint and warm fomentations. In the second group the treatment is clear and imperative ; the fluid must be removed and the joint freely irrigated either with carbolic lotion—1 in 100—or with solution of biniodide of mercury—1 in 1,000. A full-sized hydrocele trocar may be used to open the joint, and irrigation may be performed through it, or an incision may be made by the side of the patella, and if the escaping fluid should prove to be purulent, the joint must be fully opened and the finger inserted to break down adhesions, copious irrigation being then carried out. In the plastic form the prognosis is very unfavourable from the tendency to form ankylosis. The best that can be done is to keep the joint for the time being at complete rest and use hot fomentations ; indeed, the pain is so great that no alternative can be thought of. When the acute stage has somewhat subsided, a succession of small blisters may be applied, and later massage may be used. In cases of prolonged inflammation, and when such a joint has become filled up with cicatricial tissue, forcible manipulation is not only useless but absolutely mischievous, being liable to rouse again the pain and activity of the process. In mild cases, however, manipulation is advisable. In the fourth group of cases the joint affection is often comparatively unimportant, being but one manifestation of a general septicæmia. In those rare cases, however, in which the septicæmia is of a milder type, opening the joint and irrigating it freely may sometimes lead to a satisfactory result.

